# Bullying Perpetration and Narcissistic Personality Traits across Adolescence: Joint Trajectories and Childhood Risk Factors

**DOI:** 10.3389/fpsyt.2020.483229

**Published:** 2020-11-10

**Authors:** Ann H. Farrell, Tracy Vaillancourt

**Affiliations:** ^1^Counselling Psychology, Faculty of Education, University of Ottawa, Ottawa, ON, Canada; ^2^School of Psychology, Faculty of Social Sciences, University of Ottawa, Ottawa, ON, Canada

**Keywords:** bullying, narcissistic personality traits, joint trajectory, hyperactivity, anxiety, frustration, adolescence

## Abstract

**Objectives:** Although there is some evidence on the longitudinal associations between bullying perpetration and narcissistic personality traits, their joint developmental trajectories across early to late adolescence are largely unknown. Accordingly, we examined the co-development of bullying perpetration and narcissistic personality traits across adolescence and examined the childhood predictors of these joint trajectories.

**Method:** Self-reports of bullying and narcissistic personality traits were assessed across 6 years of adolescence from Grade 7 (i.e., age 13) to Grade 12 (i.e., age 18) in a sample of 616 Canadian adolescents and childhood predictors were assessed in Grades 5 and 6.

**Results:** As predicted, latent class growth analyses demonstrated that most adolescents were reflected in a trajectory of low decreasing bullying (82.0%) and a smaller group followed a moderate stable trajectory of bullying (18.0%). The majority of adolescents followed a moderate stable trajectory of narcissistic traits (56.3%), followed by a high increasing trajectory of narcissistic traits (22.8%), and a low decreasing trajectory of narcissistic traits (20.9%). Six percent of adolescents followed a high-risk dual trajectory of moderate stable bullying and high increasing narcissistic traits (high-risk group). Also as predicted, higher hyperactivity, higher frustration, and lower anxiety in childhood differentiated the high-risk group from a low-risk group (low decreasing bullying and low decreasing narcissistic traits; 19.0%). Higher childhood hyperactivity also differentiated a group of adolescents who followed a trajectory of moderate stable bullying and moderate stable narcissistic traits (10.0%) from the low-risk group. Results showed that moderate stable bullying was a better indicator of high increasing and moderate stable trajectories of narcissistic personality traits than the reverse.

**Conclusions:** Findings suggest adolescence is a time when personality and bullying reflect dynamic and heterogeneous development. Early intervention of childhood risk factors may help prevent a high-risk developmental course of bullying and narcissistic personality traits across adolescence.

## Introduction

Bullying is a significant social problem that affects up to 30% of youth and has demonstrated heterogeneous developmental pathways (1). Research from a person-centered approach has shown that the majority of youth follow a developmental trajectory pattern of low stable or decreasing bullying perpetration (approximately 42–87%), with a smaller proportion of youth following high or moderate stable or increasing trajectories [approximately 7–16% (2–5)]. Some researchers have also found evidence for a third and/or fourth group of youth following moderate stable or decreasing levels of bullying between the high and low groups [e.g., 11–35%; (4, 5)]. Identifying different subgroups of youth at risk for engaging in particular patterns of bullying can be helpful to tailor prevention and intervention efforts to adolescents. However, the joint trajectory patterns with individual differences are largely unknown. Developmental patterns of antisocial personality traits like narcissism could provide significant insight on the developmental course of bullying and youth that are at risk [e.g., (6, 7)]. Accordingly, the joint trajectories of bullying perpetration and narcissistic personality traits and their childhood predictors were examined in this study.

Bullying is a behavior that is affected by developmental and social–ecological contextual processes. From a developmental framework, bullying is a form of aggressive behavior used by children and adolescents within a power imbalance to intentionally hurt others (8, 9). This behavior peaks between early to middle adolescence, a developmental period that coincides with important biological (i.e., puberty), cognitive, psychological, and social changes (9, 10). Bullying may be one means for youth to navigate and adapt to changes such as the transition to high school, increased number of peers, and growing interest in romantic partners (11, 12). Indeed, pure bullying perpetration (i.e., engaging in bullying, but not being victimized) has been associated with important social resources such as higher social status and peer-perceived popularity (5, 13–15), dominance [e.g., (16)], power [e.g., (15)], and a greater number of dating and/or sexual partners (17, 18). Bullying can also be considered a behavior influenced by ecological contextual processes, as not all individuals use bullying behavior.

According to the ecological theory (19, 20), there are multiple nested systems varying from proximate (e.g., individual characteristics, personality) to distal (e.g., community factors, culture) that can affect development. Personality traits are important individual characteristics reflecting ways of thinking and feeling that can influence adolescent bullying perpetration directly [e.g., (21)] and indirectly by working alongside broader ecological contexts at home, school, or in the community [e.g., (22, 23)]. Bullying perpetration has been concurrently associated with personality traits reflecting antisocial tendencies including higher levels of psychopathy-linked narcissism (24) and higher levels of narcissistic exploitation (6). Children and youth who have a tendency to be exploitative and have a sense of entitlement can intentionally harm peers who they feel that they have more power over. Tendencies to be exploitative could facilitate the pursuit of bullying over time to obtain status and resources that reinforce an inflated self-image [e.g., (25, 26)]. These cross-sectional studies highlight the need for longitudinal research to determine the developmental course of antisocial personality traits alongside the development of bullying.

Personality traits are based in genetic variations, and across development, most individuals preserve their rank-order stability [i.e., rank from highest to lowest relative to all individuals (27)]. Personality research on adult samples also indicates that average levels of traits that reflect maturation such as agreeableness, conscientiousness, and emotional stability typically rise across the lifespan [e.g., (28)]. However, evidence suggests that adolescence is a developmental period when average levels of these three personality traits can drop as a result of biological, social, and psychological changes (29–31). Given the theoretical negative association between agreeableness and narcissism [e.g., (32, 33)], early adolescence is an ideal time to start examining the development of narcissism (31).

The development of moderate levels of narcissism are linked to a healthy self-worth and a positive self-concept, but higher levels of this trait reflect a sense of grandiosity, superiority, and entitlement (6, 33, 34). High levels of narcissism can also be characterized by a tendency to easily feel vulnerable and threatened when this self-view is challenged by others (25, 26, 35–37). Starting around age 8, children's developmentally normative tendencies to overestimate their own abilities begin to diminish, yet a desire for maintaining a positive self-view is evident (33, 38). During adolescence, there is some evidence suggesting variability in narcissism. In one longitudinal study, overall trends of mother-rated narcissism of children across 4 years starting at age 10 primarily reflected stability for the overall sample, but showed significant variability in individual growth trajectories [i.e., (39)]. Researchers have also begun to examine longitudinally narcissism with bullying perpetration. In one study based on a sample of youth between the ages of 11 and 13, Fanti and Henrich (34) found that baseline levels of higher narcissism and lower self-esteem predicted bullying perpetration across 1 year. In another study, Fanti and Kimonis (40) found that initial levels of narcissism were positively associated with high stable levels of bullying across 3 years of early to middle adolescence. These studies demonstrate that there can be variability in narcissism and its association with bullying but a more complete understanding of the temporal sequencing of narcissism and bullying across adolescence requires the examination of their joint trajectories.

To our knowledge, the joint trajectories of narcissism and bullying have been examined in only one study. In a sample of 393 youth followed annually across three waves starting at age 10, Reijntjes et al. (7) found four trajectory patterns of total bullying (i.e., composite of direct and indirect bullying). The majority of youth reflected a low stable pattern of bullying (37.2%), followed by an average stable pattern (27.8%), a moderate stable pattern (24.0%), and a high stable pattern (11.0%). Three trajectory groups of narcissism were found, with the largest being a medium stable group (46.8%), followed by a low stable group (43.5%), then a high stable group (9.4%). For each gender, the joint trajectories of narcissism with direct, indirect, and total bullying were examined. The majority of boys followed trajectory groups of low bullying, with low to medium narcissism (19–28% depending on the form of bullying examined), whereas the majority of girls followed trajectory groups of low bullying with low narcissism (26–42%). A small number of boys reflected high-risk joint trajectory patterns that followed high bullying and high narcissism (3–6%) and no girls followed high-risk joint trajectory patterns. In sum, Reijntjes et al. found that boys who followed the highest trajectory of narcissism were more likely to also follow a trajectory of high bullying; whereas boys following the high bullying trajectory were equally likely to follow the three narcissism trajectories. There were also a substantial number of boys who displayed high trajectories of bullying, but not narcissism. These findings suggest that high narcissism is one of many risk factors for bullying.

In the present study, we wanted to extend the study by Reijntjes et al. (7) by examining the joint developmental trajectories of bullying and narcissistic personality traits across a longer time span from early to late adolescence. Knowing the developmental pattern of narcissistic traits and bullying perpetration could help determine whether targeting cognitive-affective processes associated with exploitative and entitled tendencies in narcissistic personality may help prevent future bullying perpetration. We also wanted to contribute novel findings regarding childhood psychological and emotional risk factors of the joint trajectories of bullying and narcissistic traits. By determining childhood predictors of high-risk joint trajectory patterns, early intervention could prevent psychological and emotional patterns from escalating into long-term bullying and narcissistic traits.

Our first objective was to examine the joint developmental trajectories of bullying perpetration and narcissistic personality traits across 6 years of adolescence, starting from Grade 7 in Canada (i.e., age 13) followed annually until Grade 12 (i.e., age 18; end of high school). Based on previous studies, we predicted to find at least two trajectories of bullying perpetration, reflecting a low stable or decreasing trajectory group and a high stable or increasing trajectory group [e.g., (2, 4, 5, 22)]. We also predicted to find at least two trajectories of narcissistic traits, reflecting a low stable or decreasing trajectory group and a high stable or increasing trajectory group [e.g., (7)]. We were primarily interested in examining high-risk joint trajectory groups characterized by high bullying and high narcissistic personality traits, or moderate and/or high bullying and narcissistic personality traits. Our second objective was to examine the temporal pattern of these two trajectories. We expected that narcissistic personality traits would more readily predict bullying perpetration than the reverse given findings by Reijntjes et al. (7), but also expected that not all youth reflecting high trajectories of bullying would be high on narcissistic personality traits, as other factors could predict bullying.

To further differentiate the high-risk group from the low-risk group (low bullying perpetration and low narcissistic personality traits), our third objective was to examine childhood predictors of the joint trajectory groups assessed in Grade 5 (i.e., age 11) and Grade 6 (i.e., age 12). Childhood psychological and emotional variables that have previously been associated with bullying were examined including hyperactivity, anxiety, frustration, and empathic concern. Bullying has been associated with traits related to childhood impulsivity and a lack of inhibitory control or conscientiousness [e.g., (21, 41)]. Evidence also links bullying with lower emotional distress such as a lack of anxiety or fear [e.g., (42, 43)], and a lack of empathic concern for others (44, 45). Difficulty with emotion regulation such as suppressing anger or frustration has also been linked with bullying (46). We predicted that these childhood psychological and emotional risk factors would differentiate youth reflecting high-risk joint trajectory patterns from their peers found in a low-risk joint trajectory group.

## Materials and Methods

### Participants

Participants were from the McMaster Teen Study, which is an on-going cohort based longitudinal study on bullying, mental health, and academic achievement. In the spring of 2008, participants were recruited from 51 randomly selected primary schools from a school district in southern Ontario, Canada. Participants were in Grade 5 at Time 1 of the study and this cohort of individuals have been followed annually by the second author until Time 13, with data collection on-going. For the longitudinal study, 875 students agreed to participate, with 703 (80.6%) actually participating in at least one of the annual follow-ups from Time 2 (Grade 6) to Time 8 (Grade 12). In Grade 5, participants had a mean age of 10.91 years (*SD* = 0.36). Participants also had a median parent reported yearly household income of $70,000-$80,000 at Time 1, which was similar to that of the city of recruitment ($76,222) and province ($70,910; http://statscan.gc.ca). To be included in the current study, participants needed to have data from at least one time point across Grade 7 to Grade 12, as these were the time points available for the variables of interest for the latent class growth models. For this analytic sample, data from Grades 5 and 6 were used as predictors of the latent class growth trajectories. This led to a final analytic sample of 616 participants (87.6% of longitudinal sample; 54.2% girls). The majority of participants were White (76.1%), had a median parent reported household income of more than $80,000, and a median completed parent education level of college diploma or trades certificate.

### Procedure

Study approval was obtained from the relevant school board. At Time 1, when participants were in Grade 5, they completed measures using paper and pencil in classrooms. In subsequent time points, each year participants had the option of completing either a paper/pencil or online version of measures in their homes. Parents of participants were interviewed over the telephone by a research assistant. Every year, parental consent and youth assent forms were collected [see (47) for additional details regarding procedure]. Ethics approval was obtained from the associated university ethics councils.

### Measures

#### Trajectories From Grade 7 to Grade 12

##### Bullying

Bullying perpetration was assessed with five self-report items from an adapted version of the widely used Olweus Bully/Victim Questionnaire (1, 8). Participants were first provided with a definition of bullying followed by the question, “Since the start of the school year (September), how often have you taken part in bullying another student?” The remaining questions assessed specific forms of bullying including physical, verbal, social and cyber bullying. A five-point scale was used to assess each item (0 = *not at all* to 4 = *many times a week*), and all items were averaged to form a composite for each grade. Higher scores indicated higher bullying perpetration. The Cronbach's alphas were 0.72 in Grade 7, 0.78 in Grade 8, 0.77 in Grade 9, 0.77 in Grade 10, 0.81 in Grade 11 and 0.80 in Grade 12.

##### Narcissistic personality traits

Narcissistic personality traits were assessed using 10 items from the Narcissistic Personality Questionnaire-Revised [NPQ-R; (48)]. This measure was developed using the Narcissistic Personality Inventory as a framework [NPI; (49, 50)]. The NPI is the most commonly used scale to assess trait narcissism in non-clinical adult samples and was developed based on the criteria for narcissistic personality disorder (51, 52). Unlike other youth measures of narcissistic personality, which were designed for higher risk youth including juvenile offenders, the NPQ-R was created to assess maladaptive trait narcissism in community-based non-clinical samples of youth.

Although this measure was developed using an Asian youth sample (ages 12–19), it has been validated in North American samples (53, 54). An example of an item includes, “I can make people believe anything I want them to.” A five-point scale was used to assess each item (0 = *not at all true of me* to 4 = *very true of me*), and all items were averaged to form a composite for each grade. Higher scores indicated higher narcissistic personality traits. The Cronbach's alphas were 0.78 in Grade 7, 0.80 in Grade 8, 0.81 in Grade 9, 0.81 in Grade 10, 0.81 in Grade 11 and 0.81 in Grade 12.

#### Childhood Predictors Assessed at Grade 5 and Grade 6

##### Emotional and psychological variables

All childhood variables were assessed in Grade 5 and Grade 6. Childhood psychological variables included hyperactivity and anxiety and were assessed using the Self-Report of Personality (SRP) form of the Behavior Assessment System for Children-2 [BASC-2; (55)]. Both hyperactivity and anxiety were comprised of items that were assessed on either a four-point scale (0 = *never* to 3 = *almost always*) or a dichotomous response (0 = *false* and 2 = *true*). Hyperactivity was comprised of eight items and a sample includes, “I often do things without thinking.” Anxiety was comprised of 13 items, but one item was omitted at the request of the school board, resulting in 12 items. A sample item includes, “I worry about little things.” Items were reverse coded where appropriate and summed for each grade adjusting for missing items (55). The Cronbach's alpha reliabilities for hyperactivity were 0.79 in Grade 5 and 0.80 in Grade 6. The scores for Grade 5 and Grade 6 were then averaged to create a composite hyperactivity score (*r* = 0.51, *p* < 0.001). The alpha reliabilities for anxiety were 0.88 in Grade 5 and 0.86 in Grade 6. The scores for Grade 5 and Grade 6 were then averaged to create a composite anxiety score (*r* = 0.51, *p* < 0.001). Higher values indicated higher hyperactivity and anxiety, respectively.

Childhood emotional variables included frustration and empathic concern. Frustration was assessed with seven items from the Early Adolescent Temperament Questionnaire-Revised (EATQ-R) self-report (56, 57). A sample item includes, “It really annoys me to wait in long lines.” Each item was rated on a five-point scale (0 = *very false* and 4 = *very true*) and averaged to create a composite for each grade. Empathic concern was assessed with seven items from the Interpersonal Reactivity Index self-report [IRI; (58)]. A sample item includes, “I am a person who cares about the feelings of others.” Each item was rated on a five-point scale (0 = *not at all like me* and 4 = *always like me*) and averaged to create a composite for each grade. The Cronbach's alpha reliabilities for frustration were 0.83 in Grade 5 and 0.79 in Grade 6. The scores for Grade 5 and Grade 6 were then averaged to create a composite frustration score (*r* = 0.33, *p* < 0.001). The Cronbach's alpha reliabilities for empathic concern were 0.85 in Grades 5 and 6. The scores for Grade 5 and Grade 6 were then averaged to create a composite score (*r* = 0.52, *p* < 0.001). Higher values indicated higher frustration and empathic concern, respectively.

##### Demographic variables

Demographic variables assessed at Time 1 were biological sex, race/ethnicity, household income, and parent education. Due to the small number of races reported, race was recoded into White (83.0%) or non-White (17.0%). Household income was reported by parents using an eight-point scale (1 ≤ *$19,999*; 2 = *$20,000–29,999*; 3 = $*30,000–39,999*; 4 = $*40,000–49,999*; 5 = $*50,000–59,999*; 6 = $*60,000–69,999*; 7 = *$70,000–$79,999*; 8 ≥ *$80,000*) and highest level of completed education was reported by parents using a five-point scale (1 = *did not complete high school*; 2 = *high school*; 3 = *college diploma or trades certificate*; 4 = *university undergraduate degree*; 5 = *university graduate degree*).

### Analytic Plan

Using MPlus version 7.4 (59), semi-parametric group-based methods were estimated through latent class growth analysis. With this procedure, the number and shapes of trajectories of bullying perpetration and narcissistic personality traits across Grade 7 to Grade 12 were examined and posterior probabilities were used to identify the probability of each participant belonging to a particular trajectory group. Full information maximum likelihood estimation was used to deal with missing values. The best fitting model was determined by examining the Bayesian information criterion [BIC; (60)], the Lo-Mendell-Rubin likelihood ratio test [LMR-LRT; (61)], the bootstrapped likelihood ratio test [BLRT; (62)], and entropy. Lower values for the BIC indicate a more parsimonious model. A lower LMR-LRT and a significant BLRT indicates that the solution is a better fit than the model with one less group. Finally, entropy ranges from 0 to 1, with values closer to 1 indicating a better fit (63–65). The final selected model was also examined for theoretical and conceptual clarity. Starting values were increased to STARTS = 200 40 and LRTSTARTS = 0 0 500 200 to prevent local solutions (63). The OPTSEED function was also used to ensure that estimates were replicated. Up to four classes were tested for both bullying and narcissistic personality traits, and the best fitting univariate trajectories were used to examine the joint trajectory models. Once the final models were selected, group membership was saved and imported into SPSS for each latent class growth trajectory process (bullying perpetration, narcissistic personality traits, and joint) to allow for examining group predictors.

Before examining the significant childhood predictors of the trajectory groups, all predictors were standardized. Participants had to have data on predictors either in Grade 5 or Grade 6 and if data were available for both grades, a mean score was computed. The core analysis involved a series of multinomial logistic regression models conducted in SPSS with the saved trajectory groups and therefore participants had to have data on trajectory groups and predictors. For each latent class growth trajectory process (bullying perpetration, narcissistic personality traits, and joint), in the first series of multinomial logistic regression models, only the demographic variables were simultaneously entered as predictor variables of group membership. This was followed by a second separate series of multinomial logistic regression models which included only the childhood emotional and psychological variables entered simultaneously as predictor variables of group membership in each latent class growth trajectory process. For the univariate trajectory groups (bullying, narcissistic personality traits), the low group was selected a priori as the reference group and contrasts between high and/or moderate groups were conducted. For the joint trajectory groups (i.e., bullying and narcissistic personality traits), we were mainly interested in the groups characterized by trajectories that were high or moderate on both bullying and narcissistic personality traits (i.e., high-risk groups). Therefore, we specified three contrasts a priori and these were the only contrasts tested: (a) high bullying/high narcissistic personality traits vs. low/low (i.e., low-risk group), (b) moderate bullying/moderate narcissistic personality traits vs. low/low, and (c) high bullying/high narcissistic personality traits vs. moderate bullying/moderate narcissistic personality traits. The Benjamini–Hochberg (BH) correction was separately applied to each multinomial regression model to control for Type 1 error in multiple testing (66). For the final set of multinomial logistic regression models, all demographic, emotional, and psychological predictors were entered simultaneously for each trajectory process.

## Results

### Missing Data

The analytic sample varied slightly based on whether bullying or narcissism was available across Grades 7 to 12. The trajectory analysis for bullying included 616 participants and the trajectory analysis for narcissistic personality traits included 615 participants. For the dual trajectory, the analytic sample included 616 participants. The analytic sample was compared against the other participants in the longitudinal portion of the study (i.e., non-analytic sample) on the demographic variables using chi-square tests for sex and race, and *t*-tests for household income, parent education, and the childhood predictors (i.e., Grade 5 and 6 composites). Compared to the non-analytic sample, participants in the analytic sample were more likely to be White, have a higher household income, and have a higher level of completed parental education (all *p* < 0.001).

### Descriptive Statistics

Means and standard deviations of bullying and narcissistic personality traits across Grades 7–12 overall and by sex are shown in Table 1. All variables demonstrated acceptable skewness and kurtosis values except for bullying in Grades 9, 11, and 12, which had kurtosis values exceeding 10, and also had extreme univariate outliers (67). Winsorizing these univariate outliers allowed us to preserve rank-ordering of these individuals, reduce the skewness and kurtosis values of the variables, and reduce the impact of these individuals on the distribution of the variables (68). Overall means revealed that bullying and narcissistic personality traits were stable as they both had significant positive intercepts, but no significant slope or quadratic terms (*p* > 0.05). There were no significant sex differences in the bullying variables, but narcissistic personality trait scores were significantly higher among boys than girls at all time points except Grade 7.

**Table 1:** Descriptive statistics for joint trajectory variables.

	**Analytic sample range**		**Boys**		**Girls**		**Test**	**Total**	
	**Min**	**Max**	***M***	***SD***	***M***	***SD***	***t***	***M***	***SD***
**Bullying perpetration**									
Grade 7	0.00	2.20	0.23	0.31	0.23	0.35	0.02	0.23	0.33
Grade 8	0.00	2.40	0.30	0.40	0.27	0.39	0.87	0.28	0.39
Grade 9	0.00	3.20	0.22	0.41	0.21	0.33	0.55	0.21	0.36
Grade 10	0.00	2.40	0.17	0.27	0.18	0.33	−0.39	0.18	0.31
Grade 11	0.00	2.40	0.15	0.28	0.17	0.33	−0.56	0.16	0.30
Grade 12	0.00	3.40	0.16	0.34	0.15	0.27	0.41	0.15	0.30
**Narcissistic personality traits**									
Grade 7	0.00	3.90	2.16	0.61	2.08	0.66	1.49	2.11	0.64
Grade 8	0.00	4.00	2.19	0.63	2.03	0.68	2.77**	2.10	0.66
Grade 9	0.00	3.90	2.21	0.62	1.98	0.71	3.85***	2.08	0.68
Grade 10	0.10	4.00	2.23	0.63	1.99	0.72	3.71***	2.09	0.69
Grade 11	0.00	3.90	2.20	0.60	2.02	0.70	2.87**	2.10	0.66
Grade 12	0.20	4.00	2.24	0.61	2.05	0.66	3.13**	2.13	0.65

*Descriptive statistics are based on analytic sample N = 616; Sex coded as 0 = boys, and 1 = girls*.

** *p < 0.01*.

*** *p < 0.001*.

Bullying and narcissistic personality traits had significant small to moderate correlations in all grades except for Grade 9 (*r* = 0.12 in Grade 7, *r* = 0.11 in Grade 8, *r* = 0.10 in Grade 10, *r* = 0.20 in Grade 11, and *r* = 0.12 in Grade 12). Bullying perpetration and narcissitic personality traits were also stable across each adjacent time point (bullying: *r* = 0.54 −0.60; narcissism: *r* = 0.50–0.74). The means and standard deviations for the childhood predictor variables before standardizing for the primary analyses were as follows: hyperactivity, *M* = 5.39, *SD* = 3.66, anxiety, *M* = 9.09, *SD* = 5.53, frustration: *M* = 2.20, *SD* = 0.72, empathic concern: *M* = 2.73, *SD* = 0.61, household income, *M* = 6.26, *SD* = 2.25, and parental education, *M* = 3.20, *SD* = 1.00.

### Developmental Trajectories

#### Bullying Perpetration

**Figure 1:**
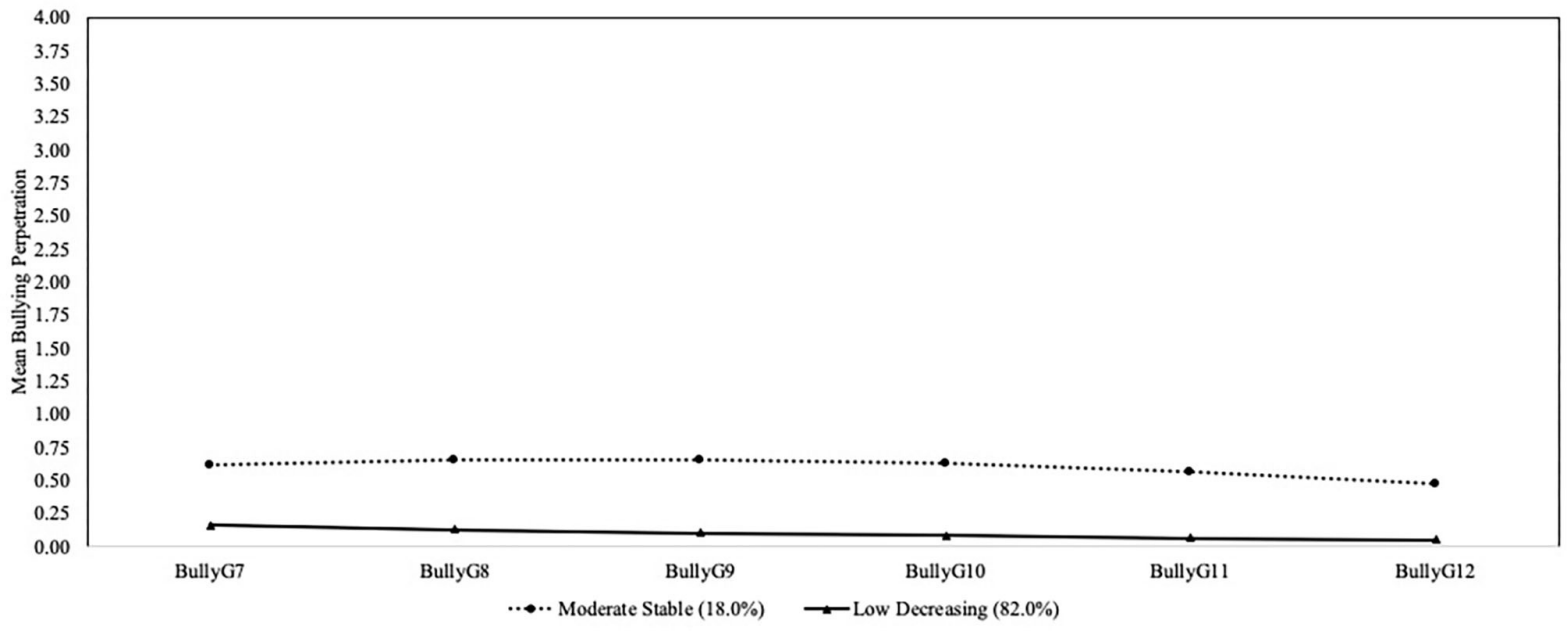
Developmental trajectories of bullying perpetration. Bully, bullying perpetration; G, grade.

The two-group solution was chosen as the final model (see Table 2 and Figure 1). Although the two-group solution had a higher BIC than the three-group solution, it was lower than the one-group solution. The entropy value for the two-group solution was also good and the same in value as the three-group solution. However, the BLRT and LMR-LRT values were significant for the two-group solution. The three- and four- group solutions did not add theoretically meaningful information. The majority of participants reflected a trajectory that started with low bullying perpetration and decreased over time (low decreasing; 82.0%, *n* = 505; 235 boys, 270 girls; intercept = 0.166, *p* < 0.001; slope = −0.034, *p* < 0.001; quadratic = 0.002, *p* = 0.139). A smaller number of the remaining participants reflected a trajectory of moderate predominately stable bullying perpetration over time, but with a slightly lower level of bullying toward the end of high school (moderate stable; 18.0%, *n* = 111; 47 boys, 64 girls; intercept = 0.619, *p* < 0.001; slope = 0.051, *p* = 0.154; quadratic = −0.016, *p* = 0.012). Participants were well-identified within their trajectory group as the posterior probabilities were 0.97 for the low decreasing group and 0.94 for the moderate stable group.

**Table 2:** Fit indices for latent class trajectory models for bullying perpetration and narcissistic personality traits.

**No. of groups**	**BIC**	**LMR-LRT**	**BLRT**	**Entropy**
**Bullying perpetration**				
1 Class	1,538.881	NA	NA	NA
2 Class	543.949	0.0003	<0.0001	0.887
3 Class	383.820	0.1935	<0.0001	0.887
4 Class	222.736	0.1617	<0.0001	0.867
**Narcissistic personality traits**				
1 Class	5,847.648	NA	NA	NA
2 Class	5,080.095	0.0001	<0.0001	0.712
3 Class	4,763.161	0.0011	<0.0001	0.746
4 Class	4,653.383	0.0594	<0.0001	0.736

*BIC, Bayesian information criterion; LMR-LRT, Lo-Mendell-Rubin likelihood ratio test; BLRT, bootstrapped likelihood ratio test*.

#### Narcissistic Personality Traits

**Figure 2:**
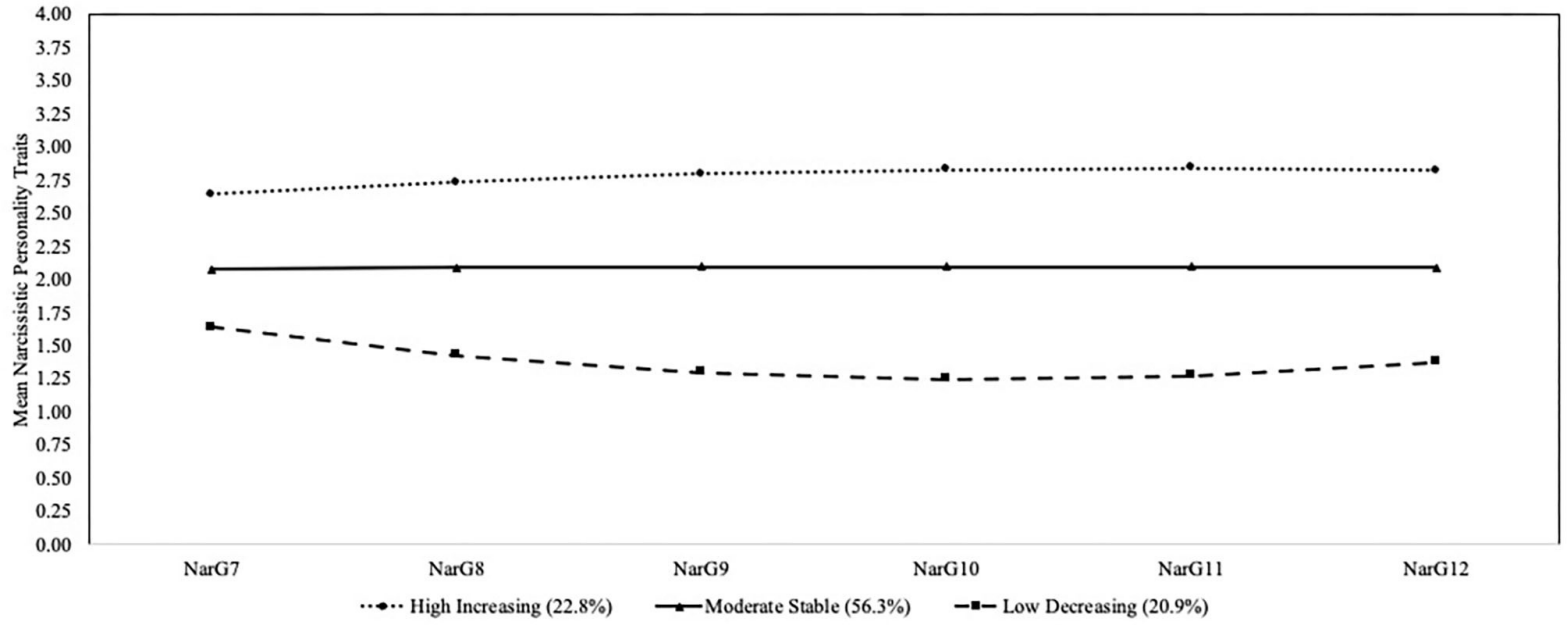
Developmental trajectories of narcissistic personality traits. Nar, narcissistic personality traits; G, grade.

The three-group solution was chosen as the final model (see Table 2 and Figure 2). Although the three-group solution had a higher BIC than the four-group solution, it was lower than the two-group solution. The entropy value for the three-group solution was better than the other solutions. The BLRT and LMR-LRT values were also significant for the three-group solution. The four-group solution did not add any theoretically meaningful information. The majority of participants reflected a trajectory that was moderate on narcissistic personality traits over time (moderate stable; 56.3%, *n* = 346; 159 boys, 187 girls; intercept = 2.078, *p* < 0.001; slope = 0.014, *p* = 0.654; quadratic = −0.002, *p* = 0.654). The next largest group of participants reflected a trajectory that started with high narcissistic traits and predominately increased over time with a slight decrease at the end of high school (high increasing; 22.8%, *n* = 140; 78 boys, 62 girls; intercept = 2.640, *p* < 0.001; slope = 0.105, *p* = 0.003; quadratic = −0.014, *p* = 0.046). The smallest group of participants reflected a trajectory that started with low narcissistic traits and predominately decreased over time with a slightly higher level toward the end of high school (low decreasing; 20.9%, *n* = 129; 44 boys, 85 girls; intercept = 1.639, *p* < 0.001; slope = −0.248, *p* < 0.001; quadratic = 0.039, *p* < 0.001). Participants were well-identified within their trajectory group as the posterior probabilities were 0.88 for the moderate stable group, 0.87 for the high increasing group, and 0.88 for the low decreasing group.

#### Joint Trajectories of Bullying Perpetration and Narcissism

There were six possible joint trajectory groups (2 × 3) with distinct developmental patterns of bullying perpetration and narcissistic personality traits. The top section of Table 3 reflects the proportion of participants in each group. The majority of participants reflected a joint trajectory pattern of low decreasing bullying and moderate stable narcissistic traits (46%, *n* = 284; 136 boys, 148 girls). The next largest group of participants reflected patterns of low decreasing trajectories of both bullying and narcissistic traits (low-risk; 19%, *n* = 115; 39 boys, 76 girls). The third largest group of participants reflected low decreasing bullying and high increasing narcissistic traits (17%, *n* = 106; 60 boys, 46 girls). Another 10% of the sample reflected trajectories of moderate stable bullying and moderate stable narcissistic traits (*n* = 63; 24 boys, 39 girls). The second smallest group of participants reflected joint trajectory patterns of moderate stable bullying and high increasing narcissistic traits (6%, *n* = 34; 18 boys, 16 girls) and the smallest group of participants reflected moderate stable bullying and low decreasing narcissistic traits (2%, *n* = 14; 5 boys, 9 girls). Therefore, the group reflecting moderate stable bullying and moderate stable narcissistic traits and the group reflecting moderate stable bullying and high increasing narcissistic traits were considered the two high-risk groups. Participants were well-identified within their trajectory group as the posterior probabilities for all joint trajectory groups were >0.81.

**Table 3:** Joint and conditional probabilities of bullying perpetration and narcissistic personality traits.

	**Narcissistic personality traits**		
**Bullying perpetration**	**High increasing**	**Moderate stable**	**Low decreasing**
**Probabilities of joint trajectory membership**^**a**^			
Moderate stable	0.06 (*n* = 34)	0.10(*n* = 63)	0.02 (*n* = 14)
Low decreasing	0.17 (*n* = 106)	0.46(*n* = 284)	0.19 (*n* = 115)
**Probabilities of bullying conditional on narcissistic traits**^**b**^			
Moderate stable	0.26	0.20	0.10
Low decreasing	0.74	0.80	0.90
**Probabilities of narcissistic traits conditional on bullying**^**c**^			
Moderate stable	0.32	0.57	0.11
Low decreasing	0.22	0.54	0.24

a *Cells total 1*.

b *Columns total 1*.

c *Rows total 1*.

The bottom section of Table 3 shows the conditional probabilities of the trajectories of bullying as a function of the trajectories of narcissistic traits, and the conditional probabilities of the trajectories of narcissistic traits as a function of the trajectories of bullying. These results suggest that a trajectory of moderate bullying was a slightly better indicator of moderate (0.57) or high (0.32) narcissistic traits than low narcissistic traits (0.11), whereas a trajectory of low bullying was a better indicator of moderate narcissistic traits (0.54) than low (0.24) and high (0.22) narcissistic traits. In contrast, all three trajectory groups of narcissistic personality traits were better indicators of low bullying than moderate bullying.

#### Childhood Predictors of Trajectory Group Membership

We examined whether there were significant differences in the proportion of boys and girls within each of the trajectory groups. There were no significant differences in boys and girls in the bullying groups, χ^2^(1) = 0.64, *p* = 0.442, but there was a significant difference in boys and girls in the narcissistic traits groups, χ^2^(2) = 12.65, *p* = 0.002, and in the joint trajectory groups, χ^2^(5) = 14.81, *p* = 0.011. There were significantly more girls (65.9%) than boys (34.1%) in the low decreasing narcissistic traits group, and more boys (55.7%) than girls (44.3%) in the high increasing narcissistic traits group. There were significantly more boys (56.6%) than girls (43.4%) in the joint trajectory group of low decreasing bullying and high increasing narcissistic traits, and more girls (66.1%) than boys (33.9%) in the joint trajectory group of low decreasing bullying and low decreasing narcissistic traits.

Contrasts for bullying groups are displayed in Table 4. The model with childhood demographic variables demonstrated that no demographic factors significantly differentiated the bullying groups. The model with childhood emotional and psychological variables indicated that higher hyperactivity significantly differentiated the moderate stable bullying perpetration group from the low decreasing bullying group, and this effect remained significant after the BH correction. When demographic, emotional, and psychological predictors were entered simultaneously, higher hyperactivity significantly differentiated the moderate group from the low group (OR = 1.463, 95% *CI* [1.122, 1.907], *p* = 0.005). Contrasts for narcissistic traits groups are displayed in Table 4. The model with childhood demographic variables demonstrated that sex significantly differentiated the high increasing narcissistic traits group from the low decreasing narcissistic traits group, with boys being more likely to predict membership in the high increasing group. This is consistent with results when examining the proportion of boys and girls in each trajectory group. The model with childhood emotional and psychological variables indicated that lower anxiety and higher frustration significantly differentiated the high increasing narcissistic traits group from the low decreasing narcissistic traits group. Lower anxiety also significantly differentiated the moderate stable group from the low decreasing group. All effects remained statistically significant after the BH correction. When all predictors were entered simultaneously, lower anxiety significantly differentiated the high (OR = 0.552, 95% *CI* [0.393, 0.774], *p* = 0.001) and moderate (OR = 0.737, 95% *CI* [0.563, 0.965], *p* = 0.026) narcissistic traits groups from the low group. Additionally, being a boy (OR = 0.513, 95% *CI* [0.290, 0.908], *p* = 0.022) and higher frustration (OR = 1.406, 95% *CI* [1.011, 1.954], *p* = 0.043) significantly differentiated the high group from the low group.

**Table 4:** Multinomial logistic regression of childhood variables predicting trajectory groups of bullying perpetration and narcissistic personality traits.

	**Trajectory group contrasts of bullying**			
	**Moderate stable vs. low decreasing**			
	***OR***	**95% CI**		
**Demographic variables**				
Sex	1.286	[0.811, 2.039]		
Race	1.370	[0.742, 2.529]		
Household income	0.888	[0.698, 1.129]		
Parent education	0.997	[0.782, 1.272]		
**Psychological and emotional variables**				
Hyperactivity	1.570*	[1.240,1.987]		
Anxiety	1.085	[0.839, 1.404]		
Frustration	1.200	[0.915, 1.573]		
Empathic concern	0.808	[0.645, 1.012]		
	**Trajectory group contrasts of narcissistic personality traits**			
	**High increasing vs. low decreasing**		**Moderate stable vs. low decreasing**	
	***OR***	**95% CI**	***OR***	**95% CI**
**Demographic variables**				
Sex	0.435*	[0.255, 0.742]	0.642	[0.405, 1.020]
Race	1.336	[0.598, 2.985]	1.527	[0.773, 3.016]
Household income	1.258	[0.942, 1.679]	1.135	[0.898, 1.435]
Parent education	1.302	[0.980, 1.730]	1.185	[0.930, 1.511]
**Psychological and emotional variables**				
Hyperactivity	1.224	[0.914, 1.634]	1.073	[0.836, 1.378]
Anxiety	0.478*	[0.349, 0.655]	0.682*	[0.534, 0.871]
Frustration	1.519*	[1.128, 2.046]	1.116	[0.874, 1.424]
Empathic concern	1.053	[0.803, 1.380]	0.897	[0.715, 1.124]

*Sex coded as 0 = boys and 1 = girls. Low decreasing trajectory group was comparison group for all contrasts*.

* *p < 0.05*.

For the joint trajectory group contrasts, the groups were first recoded into two dependent variables to allow for contrasting only the groups of interest. In the first dependent variable, the moderate bullying/moderate narcissistic traits group was coded as 1 (high-risk group 1), the moderate/high group was coded as 2 (high-risk group 2), and the low/low group was coded as 3 (low-risk comparison group). In the second dependent variable, the moderate/high group was coded as 1 and the moderate/moderate group was coded as 2, with the latter group assigned as the comparison group. Contrasts for joint trajectory groups are displayed in Table 5. The model with childhood demographic variables demonstrated that no demographic factors significantly differentiated any of the groups. The model with childhood emotional and psychological variables indicated that higher hyperactivity, lower anxiety, and higher frustration significantly differentiated the moderate bullying and high narcissistic traits group from the low-risk group, whereas higher hyperactivity and lower empathic concern significantly differentiated the moderate bullying and moderate narcissistic traits group from the low-risk group. None of the variables significantly differentiated the two high-risk groups from one another. All effects remained statistically significant after the BH correction, except for the effect of empathic concern. When all predictors were entered simultaneously, lower anxiety significantly differentiated the moderate bullying and high narcissistic traits group from the low-risk group (OR = 0.508, 95% *CI* [0.278, 0.927], *p* = 0.027). In addition, higher hyperactivity (OR = 1.620, 95% *CI* [1.048, 2.504], *p* = 0.030) and lower empathic concern (OR = 0.636, 95% *CI* [0.426, 0.950], *p* = 0.027) significantly differentiated the moderate bullying and moderate narcissistic traits group from the low-risk group. Considering Chen et al.'s (69) criteria for effect sizes of odds ratios (i.e., small = 1.68, medium = 3.47, large = 6.71), all significant odds ratios reflected small effect sizes.

**Table 5:** Multinomial logistic regression of childhood variables predicting joint trajectory groups of bullying perpetration and narcissistic personality traits.

	**Joint trajectory group contrasts**					
	**MB/HN vs. LB/LN**^****b****^		**MB/MN vs. LB/LN**^****b****^		**MB/HN vs. MB/MN**^****c****^	
	***OR***	**95% CI**	***OR***	**95% CI**	***OR***	**95% CI**
**Demographic variables**						
Sex	0.545	[0.234, 1.269]	0.949	[0.461, 1.954]	0.550	[0.211, 1.433]
Race	1.994	[0.593, 6.707]	1.937	[0.718, 5.231]	1.042	[0.280, 3.882]
Household income	1.456	[0.852, 2.487]	0.833	[0.575, 1.209]	1.785	[0.980, 3.251]
Parent education	1.204	[0.758, 1.912]	1.262	[0.853, 1.868]	0.885	[0.520, 1.504]
**Psychological and emotional variables**						
Hyperactivity	1.686*	[1.067, 2.665]	1.567*	[1.069, 2.297]	1.090	[0.639, 1.859]
Anxiety	0.466*	[0.266, 0.818]	0.831	[0.555, 1.245]	0.573	[0.314, 1.048]
Frustration	1.987*	[1.115, 3.540]	1.382	[0.883, 2.164]	1.468	[0.742, 2.901]
Empathic concern	1.050	[0.665, 1.658]	0.684^a^	[0.479, 0.976]	1.461	[0.914, 2.335]

*MB/HN, moderate stable bullying/high increasing narcissistic personality traits; LB/LN, low decreasing bullying/low decreasing narcissistic personality traits; MB/MN, moderate stable bullying/moderate stable narcissistic personality traits; sex coded as 0 = boys and 1 = girls*.

a *Non-significant after Benjamini–Hochberg correction*.

b *LB/LN was comparison group in contrast*.

c *MB/MN was comparison group in contrast*.

* *p < 0.05*.

## Discussion

The joint developmental trajectories of bullying perpetration and narcissistic personality traits were examined across 6 years of adolescence from Grade 7 to the end of high school in Grade 12. We extended Reijntjes et al.'s (7) findings by examining these joint trajectories across a longer time span starting from early to late adolescence and examining childhood predictors of the trajectories.

### Trajectories of Bullying Perpetration and Narcissistic Personality Traits

When examining trajectories of bullying perpetration alone, we found the predicted two group solution. The majority of youth reflected a low decreasing bullying trajectory (82.0%). Although the second group was higher on bullying than the low group, mean levels across the time points reflected a moderate stable trajectory (18.0%). These two groups are generally consistent with previous findings on trajectories of bullying [e.g., (2, 4, 5, 22)]. Bullying appears to be a developmentally salient form of aggressive behavior that is prevalent during the transition from early to middle adolescence as adolescents attempt to navigate social networks (9–11). The small number of youth engaging in continued moderate levels of bullying indicates that individual development can be dependent on transactions with multiple ecological contexts for some adolescents, with one of these contexts being individual differences in narcissistic personality traits [e.g., (19)].

When examining trajectories of narcissistic personality traits alone, we found three trajectory groups. The majority of participants reflected a trajectory of moderate stable narcissistic personality traits (56.3%), with the remaining youth split across the predicted low decreasing (20.9%) and high increasing (22.8%) groups. The moderate stable trajectory group indicates that the majority of youth reflect a generally positive and realistic self-concept. The high increasing group reflects a smaller proportion of adolescents who begin to display rising levels of grandiosity, superiority, and exploitative tendencies (6, 34, 38). Researchers have noted that despite theoretical proposals, there has yet to be much empirical evidence for mean level increases in narcissism during adolescence (70). One reason that we may have found significant changes in narcissistic personality traits is that we had twice the number of assessment periods compared to Reijntjes et al. (7). The six assessment periods allowed for the examination of quadratic change across the full range of adolescence, which is difficult to identify with three assessment occasions. The trajectories of narcissistic traits found in our study support adolescence as an important period of personality variability and development (29–31, 38, 39, 71). This assertion was further evident in our joint trajectory findings.

Of the six possible joint trajectory groups, our primary interest was in adolescents comprising the groups deemed to follow high-risk dual trajectories. We found that 6% of adolescents reflected a trajectory pattern of moderate stable bullying and high increasing narcissistic personality traits and 10% of adolescents reflected a trajectory pattern of moderate stable bullying and moderate stable narcissistic personality traits. We also found that 19% of adolescents reflected a trajectory pattern of low-risk (i.e., low stable bullying and narcissistic traits). These prevalence rates are somewhat consistent with findings by Reijntjes et al. (7) who found that depending on the form of bullying (i.e., indirect or direct), 3–6% of adolescent boys reflected trajectories of high bullying and high narcissism and 19-42% of adolescent boys and girls reflected low-risk joint trajectories.

The trajectory of moderate bullying was a better indicator of moderate or high narcissistic traits than the reverse. All three trajectories of narcissistic traits were better indicators of low bullying rather than moderate bullying. Only 2% of adolescents were moderate on bullying and low on narcissistic traits whereas 17% of adolescents were high on narcissistic traits and low on bullying. These results were in contrast to our predictions and findings by Reijntjes et al. (7) as these researchers found that boys who displayed high narcissism were more likely to follow trajectories of high bullying, whereas boys who displayed high bullying were equally likely to follow the three narcissism trajectories. It is possible that our findings were due to the low frequency of moderate bullying relative to low bullying and can also suggest that bullying is one of many behavioral manifestations of adolescent narcissistic personality traits [e.g., (13, 15, 25, 34)]. Further differences in these trajectory groups are evident in childhood predictors.

### Psychological and Emotional Predictors of Trajectory Groups

For the individual trajectories of bullying perpetration, hyperactivity was the only significant predictor. Youth demonstrating moderate stable bullying seem to have difficulty regulating behavior, with one form of behavior being bullying [e.g., (21, 41)]. For the individual trajectories of narcissistic personality traits, lower anxiety and higher frustration significantly differentiated membership in the high increasing group from the low decreasing group, and lower anxiety significantly differentiated membership in the moderate stable group from the low decreasing group. Our findings with anxiety have previously been supported and indicate that children who are less worried about, sensitive to, or fearful of others can be higher on narcissism [e.g., (72, 73)]. The finding with frustration has been supported in evidence linking characteristics of adolescent psychopathy, a correlate of narcissism, with lower agreeableness [e.g., (32, 74, 75)]. Youth who are easily frustrated and irritated by others could develop a sense of superiority over these peers. Sex significantly differentiated the group characterized by high increasing narcissistic traits from the group characterized by low decreasing narcissistic traits, with more boys than girls in the high group. Researchers have previously found gender differences in meta-analyses regarding some aspects and forms of narcissism but not others (76).

Childhood hyperactivity differentiated both of the high-risk joint trajectory groups from the low-risk joint trajectory group and had the largest effect relative to the other childhood predictors. Childhood anxiety and frustration also differentiated the group reflecting moderate stable bullying and high increasing narcissistic traits from the group reflecting low-risk patterns. Empathic concern additionally differentiated the group reflecting moderate stable bullying and moderate stable narcissistic traits from the group reflecting low-risk patterns prior to correcting for multiple testing, and remained significant when all childhood predictors were entered simultaneously. Previously, lower anxiety has been associated with higher antisocial tendencies including callous-unemotional traits, which could indicate lower sensitivity or care for others and a lack of fear for negative consequences [e.g., (77, 78)]. In addition to low anxiety, difficulty regulating behavioral impulses (i.e., hyperactivity), potentially during frustrating interactions with peers, could also contribute to youth developing increasing feelings of superiority and entitlement over peers. Accordingly, a moderate stable trajectory of bullying perpetration can be a behavioral indication of these early risk factors. Engaging in bullying within the context of a power imbalance is likely to further reinforce the development of superior, entitled, and narcissistic self-perceptions across adolescence. Finally, despite significant differences in the proportion of boys and girls in the low-risk group (i.e., more girls than boys), the demographic variables did not significantly differentiate the joint trajectory groups, indicating that groups were relatively similar across sex, race, and socioeconomic status.

### Limitations

There were some limitations to this study. First, all measures were self-report and subject to shared-method variance. The inclusion of additional informants such as peer-rated bullying may help reduces these biases [e.g., (79)]. However, self-reports can be valid in revealing underlying motivations of bullying perpetration that are not as easily assessed by observers (80). Second, the joint trajectory design allowed for examining the dynamics between narcissistic traits and bullying, but did not allow us to know if one causes the other. It is also possible that psychological and emotional difficulties are outcomes of the joint trajectory groups. Third, although our sample size was large, it resulted in some joint trajectory groups having smaller cell sizes. This could have underpowered our ability to find effects with our high-risk groups (*n* = 34 for the group reflecting moderate bullying and high narcissistic traits, and *n* = 63 for the group reflecting moderate bullying and moderate narcissistic traits). We also did not find a high bullying trajectory group which could have been a result of participants underreporting bullying perpetration when using self-reports. Larger sample sizes could help increase the ability to further identify individuals at-risk. Fourth, we used the Narcissistic Personality Questionnaire-Revised to assess narcissistic personality traits, which includes exploitation and superiority subscales (48). Reijntjes et al. (7) used the Childhood Narcissism Scale (33), which assesses a general construct of narcissism, and other researchers such as Fanti and Kimonis (40) have used the Antisocial Process Screening Device, which captures narcissism that co-occurs with psychopathic traits and was designed for higher-risk samples including juvenile offenders (81). We are unable to make direct comparisons of our conclusions on narcissistic traits and bullying with previous researchers' findings because we used a different measure. Researchers can investigate whether results are replicated across measures.

### Implications and Conclusions

Our findings provide support for the developmental and ecological frameworks of bullying and provide several novel contributions. First, our results revealed that a small proportion of individuals who continue to use bullying across adolescence were likely to also demonstrate high increasing or moderate and stable narcissistic personality traits. This finding suggests that addressing cognitions and attitudes related to entitlement, superiority, and exploitation can help reduce bullying behavior. Second, we found significant changes in the high increasing and low decreasing trajectories of narcissistic personality traits. Adolescence has been suggested to be an important developmental period for personality development, yet limited empirical evidence demonstrates these mean level changes [e.g., (31, 70)]. Our findings support adolescence as a malleable developmental period for narcissistic personality traits. Fourth, our results indicate that childhood psychological and emotional characteristics can predict high-risk trajectories of adolescent bullying and narcissistic traits. Intervening early signs of difficulty with behavioral and emotion regulation and a lack of sensitivity or care for others may be key methods of preventing the development of bullying and narcissistic traits in the long-term. Additional longitudinal studies examining the development of bullying and narcissistic traits can further help reveal developmental continuity and change across the lifespan, important predictors and outcomes, and critical periods for intervention.

## Data Availability Statement

The datasets generated for this study are available on request to the corresponding author.

## Ethics Statement

The studies involving human participants were reviewed and approved by the McMaster University Research Ethics Board and the University of Ottawa Office of Research Ethics and Integrity. Written informed consent to participate in this study was provided by the participants' legal guardian/next of kin.

## Author Contributions

AF and TV created the current study idea. AF performed the statistical analyses and drafted the manuscript. TV is the principal investigator of the broader longitudinal study and also helped draft the manuscript. All authors contributed to the final manuscript.

## Conflict of Interest

The authors declare that the research was conducted in the absence of any commercial or financial relationships that could be construed as a potential conflict of interest.

## References

[1] Vaillancourt T Trinh V McDougall P Duku E Cunningham L Cunningham C Optimizing population screening of bullying in school-aged children. J Sch Viol. (2010) 9:233–50. 10.1080/15388220.2010.483182

[2] Barker ED Arseneault L Brendgen M Fontaine N Maughan B. Joint development of bullying and victimization in adolescence: relations to delinquency and self-harm. J Am Acad Child Adolesc Psychiatry. (2008) 47:1030–8. 10.1097/CHI.ObO13e31817eec98 18665001

[3] Haltigan JD Vaillancourt T. Joint trajectories of bullying and peer victimization across elementary and middle school and associations with symptoms of psychopathology. Dev Psychol. (2014) 50:2426–36. 10.1037/a0038030 25313592

[4] Pepler D Jiang D Craig W Connolly J. Developmental trajectories of bullying and associated factors. Child Dev. (2008) 79:325–38. 10.1111/j.14678624.2007.01128.x 18366426

[5] Reijntjes A Vermande M Goossens FA Olthof T Van de Schoot R Aleva L. Costs and benefits of bullying in the context of the peer group: a three wave longitudinal analysis. J Abnorm Child Psychol. (2013) 41:1217–29. 10.1007/s10802-013-9759-3 23686239

[6] Ang RP Ong EY Lim JC Lim EW From narcissistic exploitativeness to bullying behavior: the mediating role of approval of aggression beliefs. Soc Dev. (2010) 19:721–35. 10.1111/j.1467-9507.2009.00557.x

[7] Reijntjes A Vermande M Thomaes S Goossens F Olthof T Aleva L. Narcissism, bullying, and social dominance in youth: a longitudinal analysis. J Abnorm Child Psychol. (2016) 44:63–74. 10.1007/s10802015-9974-1 25640909 PMC4715128

[8] Olweus D The Revised Olweus Bully/Victim Questionnaire. Bergen, Norway: Research Center for Health Promotion, University of Bergen (1996).

[9] Pepler DJ Craig WM Connolly JA Yuile A McMaster L Jiang DP A developmental perspective on bullying. Aggress Behav. (2006) 32:376–84. 10.1002/ab.20136

[10] Wang W Brittain H McDougall P Vaillancourt T Bullying and school transition: context or development? Child Abuse Negl. (2015) 51:237–48. 10.1016/j.chiabu.2015.10.004 26522183

[11] Volk AA Camilleri JA Dane AV Marini ZA. Is adolescent bullying an evolutionary adaptation? Aggress Behav. (2012) 38:222–38. 10.1002/ab.21418 22331629

[12] Volk AA Dane AV Marini ZA What is bullying? Dev Rev. (2014) 34:327–43. 10.1016/j.dr.2014.09.001

[13] Sentse M Veenstra R Kiuru N Salmivalli C. A longitudinal multilevel study of individual characteristics and classroom norms in explaining bullying behavior. J Abnorm Child Psychol. (2015) 43:943–55. 10.1007/s10802-014-9949-7 25370007

[14] Sijtsema JJ Veenstra R Lindenberg S Salmivalli C. Empirical test of bullies' status goals: Assessing direct goals, aggression, and prestige. Aggress Behav. (2009) 35:57–67. 10.1002/ab.20282 18925635

[15] Vaillancourt T Hymel S McDougall P Bullying is power. J Appl Sch Psychol. (2003) 19:157–76. 10.1300/J008v19n02_10

[16] Reijntjes A Vermande M Goossens FA Olthof T van de Schoot R Aleva L. Developmental trajectories of bullying and social dominance in youth. Child Abuse Neglect. (2013) 37:224–34. 10.1016/j.chiabu.2012.12.004 23332296

[17] Connolly J Pepler D Craig W Taradash A. Dating experiences of bullies in early adolescence. Child Maltreat. (2000) 5:299–310. 10.1177/1077559500005004002 11232258

[18] Volk AA Dane AV Marini ZA Vaillancourt T Adolescent bullying, dating, and mating: testing an evolutionary hypothesis. Evol Psychol. (2015) 13:1–11. 10.1177/1474704915613909 37924199 PMC10426866

[19] Bronfenbrenner U. The Ecology of Human Development: Experiments by Nature and Design. Cambridge, MA: Harvard University Press (1979).

[20] Bronfenbrenner U Morris PA. The bioecological model of human development. In Lerner RM, editor. Theoretical Models of Human Development. Volume 1 of Handbook of Child Psychology. 6th ed. Editors-in Chief: Damon W, Lerner RM. Hoboken, NJ: Wiley (2006). p. 793–828.

[21] Mitsopoulou E Giovazolias T Personality traits, empathy and bullying behavior: a meta-analytic approach. Aggress Violent Behav. (2015) 21:61–72. 10.1016/j.avb.2015.01.007

[22] Espelage DL van Ryzin MJ Holt MK Trajectories of bully perpetration across early adolescence: Static risk factors, dynamic covariates, and longitudinal outcomes. Psychol Violence. (2018) 8:141–50. 10.1037/vio0000095

[23] Farrell AH Volk AA Vaillancourt T Empathy, exploitation, and adolescent bullying perpetration: a longitudinal social–ecological investigation. J Psychopathol Behav Assess. (2020) 42:436–49. 10.1007/s10862-019-09767-6

[24] Stellwagen KK Kerig PK. Ringleader bullying: association with psychopathic narcissism and theory of mind among child psychiatric inpatients. Child Psychiatry Hum Dev. (2013) 44:612–20. 10.1007/s10578-012-0355-5 23271677

[25] Salmivalli C Feeling good about oneself, being bad to others? Aggress Violent Behav. (2001) 6:375–93. 10.1016/S1359-1789(00)00012-4

[26] Salmivalli C Kaukiainen A Kaistaniemi L Lagerspetz KM Self-evaluated self- esteem, peer-evaluated self-esteem, and defensive egotism as predictors of adolescents' participation in bullying situations. Pers Soc Psychol Bull. (1999) 25:1268–78. 10.1177/0146167299258008

[27] Roberts BW DelVecchio WF. The rank-order consistency of personality traits from childhood to old age: a quantitative review of longitudinal studies. Psychol Bull. (2000) 126:3–25. 10.1037/00335-2909.126.1.3 10668348

[28] Roberts BW Walton KE Viechtbauer W. Patterns of mean-level change in personality traits across the life course: a meta-analysis of longitudinal studies. Psychol Bull. (2006) 132:1–25. 10.1037/0033-2909.132.1.1 16435954

[29] Caspi A Roberts BW Shiner RL. Personality development: stability and change. Ann Rev Psychol. (2005) 56:453–84. 10.1146/annurev.psych.55.090902.141913 15709943

[30] Denissen JA Van Aken MG Penke L Wood D Self-regulation underlies temperament and personality: An integrative developmental framework. Child Dev Perspect. (2013) 7:255–60. 10.1111/cdep.12050

[31] Soto CJ Tackett JL Personality traits in childhood and adolescence: Structure, development, and outcomes. Curr Dir Psychol Sci. (2015) 24:358–62. 10.1177/0963721415589345

[32] Tackett JL Mackrell S Emerging personality in childhood and adolescence: implications for the development of narcissism and Machiavellianism. In: Barry CT, Kerig PK, Stellwagen KK, Barry TD, editors. Narcissism and Machiavellianism in Youth: Implications for the Development of Adaptive and Maladaptive Behavior. Washington, DC: American Psychological Association (2011). p. 11–23.

[33] Thomaes S Stegge H Bushman BJ Olthof T Denissen J. Development and validation of the Childhood Narcissism Scale. J Person Assess. (2008) 90:382–91. 10.1080/00223890802108162 18584447

[34] Fanti KA Henrich CC Effects of self-esteem and narcissism on bullying and victimization during early adolescence. J Early Adolesc. (2015) 35:5–29. 10.1177/0272431613519498

[35] Baumeister RF Bushman BJ Campbell WK Self-esteem, narcissism, and aggression: Does violence result from low self-esteem or from threatened egotism? Curr Dir Psychol Sci. (2000) 9:26–9. 10.1111/14678721.00053

[36] Miller JD Campbell WK Pilkonis PA. Narcissistic personality disorder: relations with distress and functional impairment. Compr Psychiatry. (2007) 48:170–7. 10.1016/j.comppsych.2006.10.003 17292708 PMC1857317

[37] Morf CC Rhodewalt F Unraveling the paradoxes of narcissism: a dynamic self-regulatory processing model. Psychol Inq. (2001) 12:177–96. 10.1207/S15327965PLI1204_1

[38] Thomaes S Bushman BJ Stegge H Olthof T. Trumping shame by blasts of noise: narcissism, self-esteem, shame, and aggression in young adolescents. Child Dev. (2008) 79:1792–801. 10.1111/j.1467-8624.2008.01226.x 19037950

[39] De Clercq B Hofmans J Vergauwe J De Fruyt F Sharp C. Developmental pathways of childhood dark traits. J Abnorm Psychol. (2017) 126:843–58. 10.1037/abn0000303 29106271

[40] Fanti KA Kimonis ER Bullying and victimization: the role of conduct problems and psychopathic traits. J Res Adolesc. (2012) 22:617–31. 10.1111/j.1532-7795.2012.00809.x

[41] Terranova AM Morris AS Boxer P. Fear reactivity and effortful control in overt and relational bullying: a six-month longitudinal study. Aggress Behav. (2008) 34:104–15. 10.1002/ab.20232 17786968

[42] van Geel M Toprak F Goemans A Zwaanswijk W Vedder P. Are youth psychopathic traits related to bullying? Child Psychiatry Hum Dev. (2017) 48:768–77. 10.1007/s10578-016-0701-0 27942914 PMC5617882

[43] Panayiotou G Fanti KA Lazarou C Fearful victims and fearless bullies? Subjective reactions to emotional imagery scenes of children involved in school aggression. Person Individ Diff. (2015) 78:29–33. 10.1016/j.paid.2015.01.011

[44] van Noorden TH Haselager GJ Cillessen AH Bukowski WM. Empathy and involvement in bullying in children and adolescents: a systematic review. J Youth Adolesc. (2015) 44:637–57. 10.1007/s10964-014-0135-6 24894581

[45] Zych I Ttofi MM Farrington DP. Empathy and callous—unemotional traits in different bullying roles: a systematic review and meta-analysis. Trauma Violence Abuse. (2019) 20:3–21. 10.1177/1524838016683456 30803395

[46] Marini ZA Dane AV Bosacki SL YLC-CURA Direct and indirect bully victims: differential psychosocial risk factors associated with adolescents involved in bullying and victimization. Aggress Behav. (2006) 32:551–69. 10.1002/ab.20155

[47] Vaillancourt T Brittain HL McDougall P Duku E. Longitudinal links between childhood peer victimization, internalizing and externalizing problems, and academic functioning: developmental cascades. J Abnorm Child Psychol. (2013) 41:1203–15. 10.1007/s10802-013-9781-5 23907699

[48] Ang RP Raine A. Reliability, validity and invariance of the Narcissistic Personality Questionnaire for Children-Revised (NPQC-R). J Psychopathol Behav Assess. (2009) 31:143–51. 10.1007/s10862-008-9112-224364423

[49] Raskin RN Hall CS A narcissistic personality inventory. Psychol Rep. (1979) 45:590 10.2466/pr0.1979.45.2.590 538183

[50] Raskin RN Hall CS. The Narcissistic Personality Inventory: alternate form reliability and further evidence of its construct validity. J Person Assess. (1981) 45:159–62. 10.1207/s15327752jpa4502_10 16370732

[51] Emmons RA. Factor analysis and construct validity of the Narcissistic Personality Inventory. J Person Assess. (1984) 48:291–300. 10.1207/s15327752jpa4803_11 16367528

[52] Raskin R Terry H. A principal-components analysis of the Narcissistic Personality Inventory and further evidence of its construct validity. J Person Soc Psychol. (1988) 54:890–902. 3379585 10.1037//0022-3514.54.5.890

[53] Loke SW Lowe PA. Validation of the narcissistic personality questionnaire for children—revised among U.S. students. Psychol Assess. (2014) 26:619–27. 10.1037/a0035442 24364423

[54] Loke SW Lowe PA Ang RP. Examination of construct bias in the Narcissistic Personality Questionnaire for children—revised across culture and gender. J Early Adolesc. (2018) 38:714–37. 10.1177/027243161769244224364423

[55] Reynolds CR Kamphaus RW. Behavior Assessment System for Children: Manual. 2nd ed. Minneapolis, MN: Pearson (2004).

[56] Capaldi DM Rothbart MK Development and validation of an early adolescent temperament measure. J Early Adolesc. (1992) 12:153–73. 10.1177/0272431692012002002

[57] Ellis LK Rothbart MK Revision of the early adolescent temperament questionnaire. In: Poster Presented at the 2001 Biennial Meeting of the Society for Research in Child Development. Minneapolis, MN (2001).

[58] Davis MH A multidimensional approach to individual differences in empathy. JSAS Catalog Selected Documents Psychol. (1980) 10:85.

[59] Muthén LK Muthén BO (1998–2017). Mplus User's Guide. 8th ed Los Angeles, CA: Muthén and Muthén.

[60] Schwartz G Estimating the dimension of a model. Ann Statist. (1978) 6:461–4.

[61] Lo Y Mendell N Rubin D Testing the number of components in a normal mixture. Biometrika. (2001) 88:767–78. 10.1093/biomet/88.3.767

[62] McLachlan G Peel D Finite Mixture Models. New York, NY: Wiley (2000).

[63] Jung T Wickrama KAS Recent advances in longitudinal data analysis in social and psychological research: an introduction to latent class growth analysis and growth mixture modeling. Soc Personal Psychol Compass. (2007) 2:302–31. 10.1111/j.1751-9004.2007.00054.x

[64] Nagin DS Group-Based Modelling of Development. Cambridge: Harvard Press (2005).

[65] Nylund KL Asparouhov T Muthén BO Deciding on the number of classes in latent class analysis and growth mixture modeling: a Monte Carlo simulation study. Struct Equ Modeling. (2007) 14:535–69. 10.1080/10705510701575396

[66] Benjamini Y Hochberg Y Controlling the false discovery rate: a practical and powerful approach to multiple testing. J R Stat Soc. Series B Methodol. (1995) 57:289–300.

[67] Kline RB Principles and Practice of Structural Equational Modeling (4th ed.). New York, NY: The Guilford Press (2016).

[68] Tabachnick BG Fidell LS Using Multivariate Statistics (6th ed.). Boston, MA: Pearson Education (2013).

[69] Chen H Cohen P Chen S How big is a big odds ratio? Commun Stat Simul Comput. (2010) 39:860–4. 10.1080/03610911003650383

[70] Hill PL Roberts BW Narcissism as a life span construct: describing fluctuations using new approaches. In: Hermann A, Brunell A, Foster J, editors. Handbook of Trait Narcissism. Cham: Springer (2018). p. 165–72.

[71] Ashton MC Lee K Age trends in HEXACO-PI-R self-reports. J Res Pers. (2016) 64:102–11. 10.1016/j.jrp.2016.08.008

[72] Calhoun GB Glaser BA Stefurak T Bradshaw CP Preliminary validation of the narcissistic personality inventory–juvenile offender. Int J Offender Ther Comp Criminol. (2000) 44:564–80. 10.1177/0306624X00445004

[73] Lau KS Marsee MA Kunimatsu MM Fassnacht GM Examining associations between narcissism, behavior problems, and anxiety in non-referred adolescents. Child and Youth Care Forum. (2011) 40:163–76. 10.1007/s10566-010-9135-1

[74] Essau CA Sasagawa S Frick PJ. Callous-unemotional traits in a community sample of adolescents. Assessment. (2006) 13:454–69. 10.1177/1073191106287354 17050915

[75] Lynam DR Caspi A Moffitt TE Raine A Loeber R Stouthamer-Loeber M. Adolescent psychopathy and the Big Five: results from two samples. J Abnorm Child Psychol. (2005) 33:431–43. 10.1007/s10648-005-5724-0 16118990

[76] Grijalva E Newman DA Tay L Donnellan MB Harms PD Robins RW. Gender differences in narcissism: a meta-analytic review. Psychol Bull. (2015) 141:261–310. 10.1037/a0038231 25546498

[77] Kahn RE Frick PJ Golmaryami FN Marsee MA. The moderating role of anxiety in the associations of callous– unemotional traits with self-report and laboratory measures of affective and cognitive empathy. J Abnorm Child Psychol. (2017) 45:583–96. 10.1007/s10802-016-0179-z 27364345

[78] Vaillancourt T Brittain H. Longitudinal associations among primary and secondary psychopathic traits, anxiety, and borderline personality disorder features across adolescence. Personal Disord. (2019) 10:354–64. 10.1037/per0000325 30628800

[79] Pellegrini AD Long JD A longitudinal study of bullying, dominance, and victimization during the transition from primary school through secondary school. Br J Dev Psychol. (2002) 20:259–89. 10.1348/2F026151002166442

[80] Volk AA Veenstra R Espelage DL So you want to study bullying? Aggress Violent Behav. (2017) 36:34–43. 10.1016/j.avb.2017.07.003

[81] Frick PJ Hare RD. Antisocial Process Screening Device: APSD. Toronto: Multi Health Systems (2001).

